# An Off-Line DPPH-GC-MS Coupling Countercurrent Chromatography Method for Screening, Identification, and Separation of Antioxidant Compounds in Essential Oil

**DOI:** 10.3390/antiox9080702

**Published:** 2020-08-03

**Authors:** Xiang Wang, Guang-Lei Zuo, Chao-Yue Wang, Hyun Yong Kim, Soon Sung Lim, Sheng-Qiang Tong

**Affiliations:** 1 College of Pharmaceutical Science, Zhejiang University of Technology, Hangzhou 310032, China; 2111807066@zjut.edu.cn (X.W.); 2111707019@zjut.edu.cn (C.-Y.W.); 2 Department of Food Science and Nutrition, Hallym University, 1 Hallymdeahak-gil, Chuncheon 24252, Korea; B16504@hallym.ac.kr (G.-L.Z.); 41310@hallym.ac.kr (H.Y.K.); 3 Institute of Korean Nutrition, Hallym University, 1 Hallymdeahak-gil, Chuncheon 24252, Korea; 4 Institute of Natural Medicine, Hallym University, 1 Hallymdeahak-gil, Chuncheon 24252, Korea

**Keywords:** antioxidant, DPPH-GC offline, HSCCC, essential oil, curcuma

## Abstract

Essential oils are an important source of natural antioxidants and multiple methods have been established for evaluation of their overall antioxidant activity, however, the antioxidant activities of their compounds are less investigated. In the present study, the hyphenation of 2,2′-diphenyl-1-picrylhydrazyl (DPPH)-gas chromatography (GC)-mass spectrometry (MS) offline and high-speed countercurrent chromatography (HSCCC) is established for efficient screening, identification, and isolation of antioxidants from essential oils and applied to the essential oil of *Curcuma wenyujin* Y.H. Chen et C. Ling. Five compounds are preliminarily screened as antioxidants using DPPH-GC according to the reduction of GC peak areas of each compound after reaction with DPPH and then identified as eucalyptol (7.66%), camphor (2.34%), *δ*-elemene (1.15%), *β*-elemene (7.10%), and curzerene (15.77%) using GC-MS. Moreover, these five compounds are isolated by HSCCC using two solvent systems, *n*-hexane-acetonitrile-ethanol (5:3:2, *v/v*) and *n*-hexane-acetonitrile-acetone (4:3:1, *v/v*), and subjected to DPPH scavenging assay. Camphor, *δ*-elemene, and *β*-elemene show weak DPPH scavenging activity, while curzerene and eucalyptol show moderate DPPH scavenging activity. Notably, a significant synergistic effect on DPPH scavenging is found between curzerene and eucalyptol. The result demonstrated that off-line DPPH-GC-MS coupling CCC is an efficient method for screening, identification, and separation of antioxidant compounds in essential oil

## 1. Introduction

Essential oils are fluid mixtures obtained from fragrant plants, which have been used in cosmetic fragrances, food flavors, and traditional medicines for several centuries [1]. Multiple beneficial effects of essential oil, such as antioxidation [2], anti-inflammation [3], antibacterial [4], and anticancer activities [5], have been investigated and applied for prevention and treatment of diseases. Moreover, some essential oils with good reducing powder and free radical scavenging capacities can be developed as food stabilizers and active packaging for protection of food from spoilage [6,7,8,9]. However, considering that essential oils are relatively safe and widely accepted by consumers [8], the study of essential oil antioxidants is still highly encouraged.

The evaluation of the antioxidant activity of essential oils (a mixture of compounds) can be easily carried out using several methods [10,11,12,13,14,15]. However, due to the lack of efficient methods for screening and separation of the potential antioxidant compounds, particular minor antioxidants, from essential oils, the antioxidant activity of the individual compounds from essential oils are usually less studied. Thin layer chromatography (TLC)-DPPH-based bioautography strategy has widely been applied for detection of the antioxidants in essential oils because its advantages of easy operation, flexibility, high sample throughput, and quick access to the localization of the antioxidant compounds in essential oils [16]. However, the co-elution of some compounds may occur due to the low resolution of TLC and, more importantly, the minor compounds are usually not detectable by TLC. In contrast, GC has satisfactory chromatography resolution and sensitivity. Therefore, combination of DPPH and GC can be a promising approach to efficiently screen antioxidant compounds from essential oils like as the good performance of DPPH-HPLC on screening of antioxidants from polar and medium polar natural products [17,18,19,20,21]. Once the antioxidant compounds are screened by DPPH-GC offline, their structures can then be identified by GC-MS.

The essential oil of *Curcuma wenyujin* Y.H. Chen et C. Ling (*C. wenyujin* Y.H. Chen et C. Ling) has a large variety of pharmacological properties, such as anti-inflammatory, anticancer, antiproliferative, antidiabetic, antihepatotoxic, anti-oxidation, antibacterial, antiviral, and insecticide, etc. [22,23,24,25,26]. *C. wenyujin* Y.H. Chen et C. Ling essential oil has a moderate antioxidant activity and the compounds it contains have well been identified [27,28], thus it is a proper material to verify the proposed DPPH-GC-MS method. Nevertheless, comparative evaluation of the antioxidant activity using pure compounds, rather than an essential oil mixture, is supposed to give a stronger verification of the developed method. Therefore, the countercurrent chromatography (CCC) separation technique, with distinct advantages of the large-scaling, total sample recovery, low risk of sample denaturation, and without irreversible adsorption [24,29,30,31], is employed to separate the screened antioxidant compounds from *C. wenyujin* Y.H. Chen et C. Ling essential oil to assess their antioxidant activity.

Overall, the aim of this study was to establish an off-line DPPH-GC-MS coupling CCC method for efficient screening, identification, and isolation of antioxidant compounds in *C. wenyujin* Y.H. Chen et C. Ling essential oil. The newly developed method is expected to further promote the study on the evaluation of the antioxidant activity of the compounds in essential oils. In addition, the potential synergistic effect of the highly active compounds was also investigated.

## 2. Materials and Methods

### 2.1. Reagents and Materials

*n*-Hexane, acetonitrile, acetone, and ethanol used for HSCCC isolation were analytical grade and bought from Samchun Pure Chemical Co., Ltd. (Pyeongtaek-si, Gyeonggi-do, South Korea). Methanol used for sample dilution and GC analysis was chromatographic grade and bought from J. T. Baker (Phillipsburg, NJ, USA). Butylated hydroxytoluene (BHT) and 1,1-diphenyl-2-picryl-hydrazyl (DPPH) were bought from Sigma-Aldrich Chemical Co. (St. Louis, MO, USA), and the fresh DPPH radical solutions was requisite and kept away from the light. Essential oil of *C. wenyujin* Y.H. Chen et C. Ling rhizome was bought from Shunming volatile oil refinery (Jiangxi, Jishui, China), which was extracted by hydrodistillation.

### 2.2. Apparatus

Liquid-liquid separation was conducted on a TBE-300C HSCCC (Tauto Biotechnique Company, Shanghai, China). The apparatus was consisted of three coiled columns connected in series (diameter of tube: 1.6 mm; total capacity: 300 mL), a 20 mL sample loop, and an Isolera FLASH purification system (Biotage, Uppsala, Sweden) as the pump, fraction collector, and UV monitor. The revolution speed was set at 850 rpm.

The essential oil was analyzed using an Agilent 6890N series GC equipped with a flame ionization detector (FID) and a HP-5 5% phenyl methyl siloxane capillary column (Agilent 19091J-413, 30 m × 0.32 mm i.d., film thickness 0.25 μm), and GC-MS was performed on the Agilent Technologies 7820A GC system combined with an Agilent 5977E series GC/MSD.

### 2.3. DPPH Radical Scavenging Assay

The DPPH radical scavenging assay was performed on an EL800 Universal Microplate reader (Bio-Tek Instruments, Inc.) using multi-well plates as a previously published method described [32]. Firstly, different concentrations of positive control BHT (1.5, 0.75, 0.375, 0.1875, and 0.094 mg/mL) and *C. wenyujin* Y.H. Chen et C. Ling essential oil (3.36, 1.68, 0.84, 0.42, and 0.21 mg/mL) were prepared. Then the DPPH solution was diluted by methanol to 32 µM as a working solution. The reaction was initiated by mixing 20 µL of sample solution with 180 µL of DPPH working solution and incubated in dark at room temperature for 30 min. A monitoring of the absorbance at 570 nm was carried out after the reaction was completed. The scavenging capacity of samples were calculated by experimental scavenging capacity (ESC) using Equation (1) as follows [33]:

(1) $$\%ESC=100-\left\{\left[\left(Abs_{sample}-Abs_{blank}\right)\times 100\right]∕Abs_{control}\right\}$$

where *Abs_sample_* is the absorbance value of the sample (DPPH solution plus antioxidant) at each time interval, *Abs_blank_* is the absorbance value of the blank (methanol plus antioxidant(s)). *Abs_control_* is the absorbance value of control (methanol plus DPPH solution).

The value of 50% inhibition (IC_50_) was calculated by the graph plotting sample concentration and inhibition percentage.

### 2.4. DPPH-GC Offline Experiment and Structure Identification by GC-MS

The *C. wenyujin* Y.H. Chen et C. Ling essential oil (20 µL, 3.36 mg/mL) was mixed with DPPH solutions with different concentrations (180 µL, 2.5, 5, 10 mg/mL), and then the mixtures were incubated in dark at room temperature for 30 min. A mixture of *C. wenyujin* Y.H. Chen et C. Ling essential oil and methanol, a solvent used for preparation of DPPH solution, was set as a control group. The mixtures were further monitored by GC using a HP-5 phenyl methyl siloxane column (320 μm × 30 m, film thickness 0.25 μm). Nitrogen was used as a carrier gas at a flow rate of 2 mL/min. The conditions were as follows: inlet temperature 65 ℃ for 2 min, 5 ℃/min to 90 ℃, 90 ℃ for 3 min, 8 ℃/min to 150 ℃, 150 ℃ for 15 min, 12 ℃/min to 180 ℃, 180 ℃ for 3 min, 16 ℃/min to 210 ℃, 210 ℃ for 3min, 20 ℃ /min to 280 ℃, 280 ℃ for 10 min. The injector temperature was held at 250 ℃ and the injection volume of sample was 1 µL with a spilt ratio at 50:1. The solvent delay carriage gases were N_2_ and H_2_. The compounds with GC peak areas decreased after reaction with DPPH solution compared with those from the control group can be assumed as DPPH free radical scavengers.

The structures of the screened antioxidants as well as other principle components were then identified by GC-MS by the Agilent Technologies 7820A GC system combined with an Agilent 5977E series GC/MSD using the same condition as descried for GC assay.

### 2.5. HSCCC Separation

#### 2.5.1. Selection of Biphasic Solvent System

The proper solvent system was determined by GC on the basis of the partition coefficients (0.2 < *K* < 5) and separation factor (*α* > 1.5) [34]. Approximately 20 µL of *C. wenyujin* Y.H. Chen et C. Ling essential oil was dissolved in a 400 µL pre-equilibrated biphasic solvent system. After a vigorous shake for three minutes and thorough equilibration for 20 min, 200 µL of upper and lower phase of biphasic solvent were analyzed by GC to obtain the peak areas *A_1_* and *A_2_* for each targeted compound, respectively. The *K* value was defined as *K* = *A_1_*/*A_2_*, and the separation factor was calculated by the equation *α* = *K*_1_/*K*_2_.

#### 2.5.2. Preparation of Solvent System and Sample Solution

Two organic solvent systems consisted of *n-*hexane-acetonitrile-ethanol (5:3:2, *v/v*) and *n*-hexane-acetonitrile-acetone (4:3:1, *v/v*) were used for HSCCC separation of target compounds from *C. wenyujin* Y.H. Chen et C. Ling essential oil. Each biphasic solvent system was prepared in a separation funnel on the basis of volume ratios, and thoroughly equilibrated after shaking vigorously for three minutes at room temperature, and then the upper and lower phase were separated as the stationary phase and mobile phase, respectively. The sample solution was prepared by dissolving 420 mg of *C. wenyujin* Y.H. Chen et C. Ling essential oil in 4 mL of solvent mixtures composed of upper phase and lower phase (1:1, *v/v*) of each solvent system.

#### 2.5.3. HSCCC Separation Procedure

Each HSCCC separation was performed by head-to-tail elution mode which used the upper layer as stationary phase and lower layer as mobile phase. The stationary phase was pumped into the column with a flowrate of 60 mL/min, and the mobile phase was pumped into column with a flowrate of 2 mL/min while the rotation speed was set at 850 rpm. After the biphasic equilibrium was established, the sample solution was loaded for separation. The wavelength of the UV detector was set at 210 nm and the fractions were collected automatically.

### 2.6. Statistical Analysis

All data of the samples were tested with three-time repeats and the results were given as mean ± standard deviation (SD). The value of IC_50_ was calculated by the plot of sample concentrations and the DPPH radical scavenging activity. The statistical analysis was performed on using ANOVA (Tukey test) by SPSS version 17.0 software (IBM, Armonk, NY, USA). *p* < 0.05 was considered statistically significant.

## 3. Results and Discussion

### 3.1. DPPH Radical Scavenging Activity

**Figure 1:**
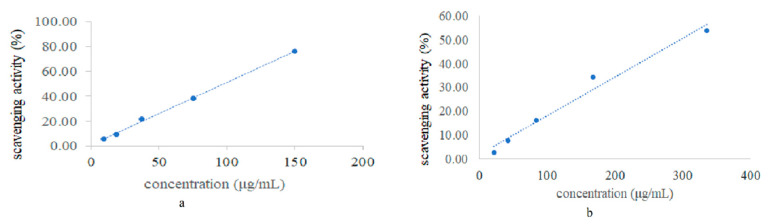
Scavenging activity of DPPH radical by BHT (**a**) and the essential oil of *Curcuma wenyujin* Y.H. Chen et C. Ling (**b**).

The antioxidant activity of *C. wenyujin* Y.H. Chen et C. Ling essential oil was determined by the DPPH radical scavenging assay. The radical scavenging activities of BHT and essential oil are shown in Figure 1. The IC_50_ was obtained by the plot of inhibition percentage and sample concentration. As shown in Table 1, the *C. wenyujin* Y.H. Chen et C. Ling essential oil showed a moderate antioxidant activity (IC_50_: 292.88 µg/mL) compared with the positive control BHT (IC_50_: 97.44 µg/mL). This result was consistent with a previous study [35].

### 3.2. Screening of Antioxidants in Curcuma wenyujin Y.H. Chen et C. Ling Essential Oil by the DPPH-GC Offline Method

The screening of antioxidants from natural product extracts with polar and medium polar polarities can be efficiently achieved using DPPH-HPLC offline [36], however, which does not apply to nonpolar sample, such as essential oil. In contrast, DPPH-TLC has widely been used to screen antioxidant compounds from essential oil, which is able to locate the antioxidants’ positions on the sprayed TLC plate via bioautography and has advantages of simplicity, high throughput, and flexibility [16]. However, the DPPH-TLC method is also criticized for its low separation resolution and low detection sensitivity, which may cause the overlap of compounds with similar polarity on the TLC plate and the missing detection of some active minor compounds [37]. Considering that GC has a high separation resolution, high detection sensitivity, easy-operation and, particularly, a combination of GC and MS can be easily achieved, a DPPH-GC offline strategy was proposed to efficiently screen antioxidants from essential oil.

**Figure 2:**
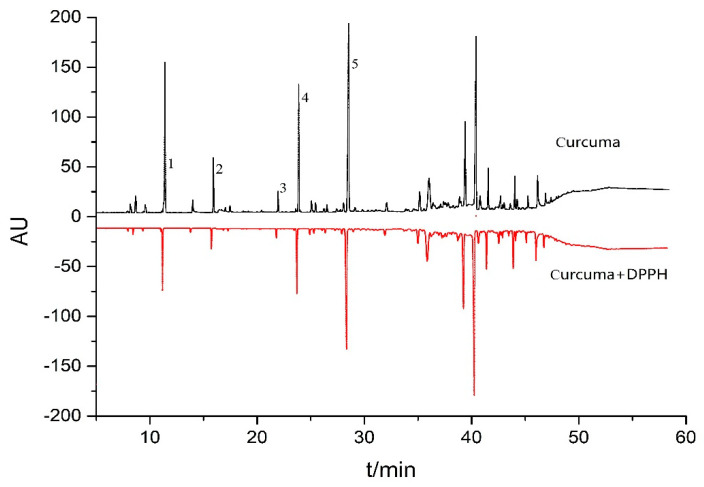
Chromatograms of the *Curcuma wenyujin* Y.H. Chen et C. Ling essential oil before and after reaction with DPPH radicals. Column: HP-5 phenyl methyl siloxane (30 m × 0.32 mm), column temperature programmed: inlet temperature 65 ℃ for 2 min, 5 ℃/min to 90 ℃, 90 ℃ for 3 min, 8 ℃/min to 150 ℃, 150 ℃ for 15 min, 12 ℃/min to 180 ℃, 180 ℃ for 3 min, 16 ℃/min to 210 ℃, 210 ℃ for 3min, 20 ℃/min to 280 ℃, 280 ℃ for 10 min, flow rate 2 mL/min, volume of injection 1 µL (1.67 mg/mL), spilt mode 50:1, injector temperature 250 ℃, carriage gas: N_2_, H_2_.

In principle, the GC peak areas of the antioxidant compounds in an essential oil will decrease after reaction with the DPPH free radical compared with those from the control group (without mixing with the DPPH free radical solution) as the HPLC-DPPH offline method is performed [18], which is able to screen the potential antioxidants from the essential oil. Accordingly, *C. wenyujin* Y.H. Chen et C. Ling essential oil, with moderate antioxidant activity, was used to test the DPPH-GC method. As indicated by Figure 2, which was obtained by mixing 20 µL of essential oil (3.36 mg/mL) and 180 µL of DPPH solution (2.5 mg/mL) after an incubation of 30 min at the temperature described in Section 2.4, the peak areas of five GC peaks, numbered by 1, 2, 3, 4, and 5, of the DPPH-treated sample were significantly decreased compared with those from the DPPH-free *C. wenyujin* Y.H. Chen et C. Ling essential oil (control group). Meanwhile, the peak areas of non-antioxidants in *C. wenyujin* Y.H. Chen et C. Ling essential oil scarcely changed before and after the reaction with DPPH free radical. Thus, five compounds marked by numbers 1–5 were preliminarily screened as potential antioxidants.

### 3.3. Determination of Off-Line Experimental Conditions

**Figure 3:**
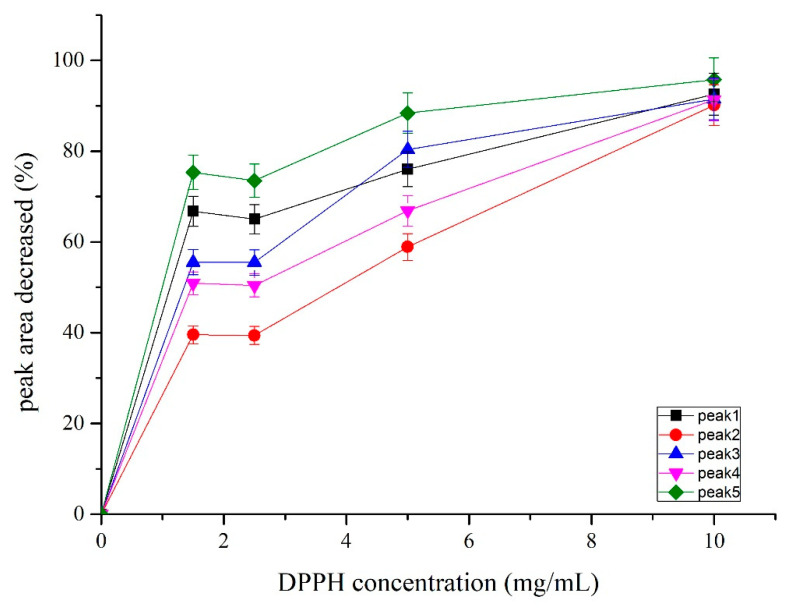
The peak areas of peaks 1–5 reduced after treated with different concentrations of DPPH methanol solution (stock concentration).

The concentrations of DPPH used for DPPH-GC offline were further studied. Four different concentrations of DPPH stock solutions, 1.25, 2.5, 5, and 10 mg/mL, were prepared and 180 µL of each DPPH solutions were respectively mixed with 20 µL of *C. wenyujin* Y.H. Chen et C. Ling essential oil (stock concentration 3.36 mg/mL) and incubated for 30 min at room temperature, as the HPLC-DPPH method was performed [18,19]. The reaction solutions were then checked by GC to measure the peak areas of the five active compounds from DPPH-containing groups and DPPH-free group. As shown in Figure 3, the peak areas of the five active compounds decreased significantly after the reaction with DPPH radicals compared with the DPPH-free group. In general, the ratios of the decreased peak areas of the five active compounds showed a DPPH dose-dependent manner and higher ratios of the decreased peak areas might indicate higher DPPH scavenging activity of the compounds tested. However, as indicated by Figure 3, too much high concentration of DPPH (e.g., 10 mg/mL) may attenuate the differences between the peak decrease ratios of high activity compounds and low activity compounds since the low activity compounds can also be attacked by an excess amount of DPPH radical, which is consistent with the result from a previous DPPH-HPLC offline study [19]. Therefore, in the present study, 1.25 mg/mL and 2.5 mg/mL of DPPH (stock concentration) are suitable concentrations used to preliminarily rank the DPPH scavenging activities of these five compounds, according to which the DPPH scavenging activities preliminarily ranked as 5 > 1 > 3 > 4 > 2.

### 3.4. Identification of the Antioxidants in Curcuma wenyujin Y.H. Chen et C. Ling Essential Oil by GC-MS

The identification of the antioxidants and other non-active compounds in *C. wenyujin* Y.H. Chen et C. Ling essential oil was carried out using GC-MS and the result was shown in Table 2. The five antioxidants screened by DPPH-GC offline assay were identified as: peak 1, eucalyptol (11.40 min, 7.66%); peak 2, camphor (15.93 min, 2.34%); peak 3, *δ*-elemene (21.97 min, 1.15%); peak 4, *β*-elemene (23.89 min, 7.10%); and peak 5, curzerene (28.52 min,15.77%). Notably, the minor compounds camphor and *δ*-elemene, which might not be detectable by TLC due to their small content in *C. wenyujin* Y.H. Chen et C. Ling essential oil (peak area ratios less than 3%), were successfully screened as antioxidants by DPPH-GC offline method. Among the five screened target compounds, curzerene and eucalyptol have previously reported to show antioxidant activity [24,38], which validates the DPPH-GC method.

### 3.5. HSCCC Separation

**Figure 4:**
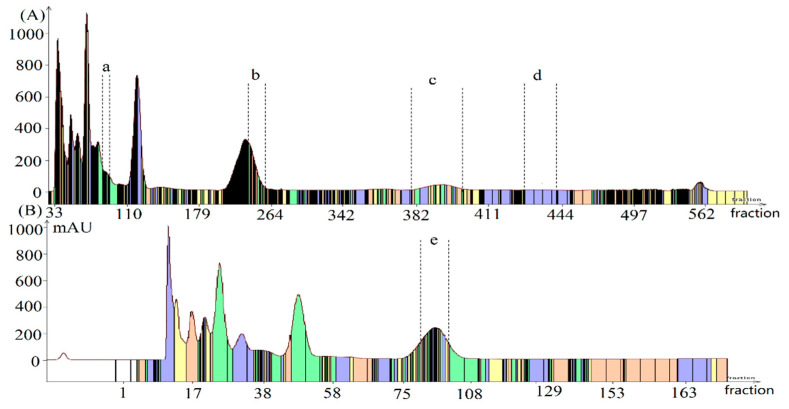
Chromatogram for separation of antioxidants from *Curcuma wenyujin* Y.H. Chen et C. Ling essential oil by countercurrent chromatography (**A**) and (**B**). Solvent system: (**A**) *n-*hexane-acetonitrile-ethanol (5:3:2, *v/v*) (**B**) *n*-hexane-acetonitrile- acetone (4:3:1, *v/v*); stationary phase: upper organic phase; mobile phase: lower organic phase; flow rate: 2 mL min^−1^; revolution speed: 850 rpm; detection wavelength: 210 nm; sample injection: (**A**) 420mg of essential oil of *Curcuma wenyujin* Y.H. Chen et C. Ling; stationary phase retention: 57% (**A**) and 66% (**B**).

HSCCC was employed to separate the five targeted antioxidants from *C. wenyujin* Y.H. Chen et C. Ling essential oil. The organic biphasic solvent systems were used because some components may degrade in aqueous solution [39]. Two solvent systems, consisting of *n*-hexane-acetonitrile-ethanol (5:3:2, *v/v*) and *n-*hexane-acetonitrile-acetone (4:3:1, *v/v*), were selected on the basic of their favorable solubility of the sample, suitable partition coefficients *K*, and separation factor α. As shown in Table 3, it was found that the *K* values of these five compounds were between 0.2–5.0 in solvent system A, and the separation factors were also acceptable. Thus solvent system *n*-hexane-acetonitrile-ethanol (5:3:2, *v/v*) was firstly used for separation of 420 mg of *C. wenyujin* Y.H. Chen et C. Ling essential oil and the separation chromatogram was shown in Figure 4A. The lower phase of *n*-hexane-acetonitrile-ethanol (5:3:2, *v/v*) was used as the mobile phase with a flowrate at 2 mL/min for 0–300 min, and the upper phase of *n*-hexane-acetonitrile-ethanol (5:3:2, *v/v*) was used as the mobile phase with a flowrate at 20 mL/min in the extrusion elution. The GC analysis indicated that four peaks with high purity corresponding to compound 2 (camphor, 3.14 mg), compound 3 (*δ*-elemene, 1.18 mg), compound 4 (*β*-elemene, 2.76 mg), and compound 5 (curzerene, 10.71 mg) were obtained. However, compound 1 was co-eluted with partial compound 5 due to their similar *K* values (*K*_1_ = 1.51, *K*_5_ = 1.30).

**Figure 5:**
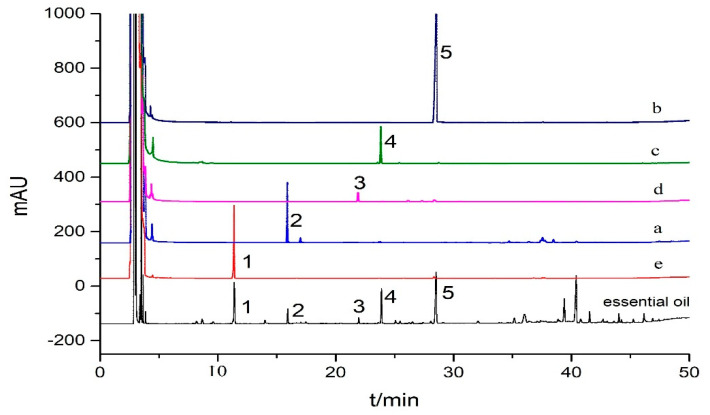
GC chromatograms of compounds separated from *Curcuma wenyujin* Y.H. Chen et C. Ling essential oil by HSCCC separation.

The solvent system of *n*-hexane-acetonitrile-acetone (4:3:1, *v/v*) was then used to separate compound 1 due to the big difference of *K* values between compound 1 (*K*_1_ = 3.15) and compound 5 (*K*_5_ = 1.84). As shown in Figure 4B and Figure 5e, a high purity (97%) of compound 1 (eucalyptol, 3.13 mg) was separated from 420 mg of *C. wenyujin* Y.H. Chen et C. Ling essential oil using this solvent system. Consequently, all of the five target compounds have been separated by running HSCCC separation two times.

### 3.6. Antioxidant Activities of the Separated Compounds and Their Potential Synergistic Effect

A comparative evaluation of the DPPH scavenging activity of the isolated compounds and *C. wenyujin* Y.H. Chen et C. Ling essential oil at 336 µg/mL (final concentration) was first carried out and the result was shown in Table 4. However, only curzerene showed a slightly higher DPPH radical scavenging activity than *C. wenyujin* Y.H. Chen et C. Ling essential oil by giving DPPH scavenging activity of 70.02% and 68.98%, respectively. Eucalyptol showed a moderate DPPH scavenging activity 47.14% but lower than *C. wenyujin* Y.H. Chen et C. Ling essential oil (68.98%), while compounds camphor, *δ*-elemene, and *β*-elemene showed very weak DPPH scavenging activity (data not shown). Considering that *C. wenyujin* Y.H. Chen et C. Ling essential oil showed higher DPPH scavenging activity than four of the purified compounds and similar activity with one purified compound, a synergistic effect was considered to exist among these compounds. Then we tested the potential synergistic effect using two relatively high active compounds, curzerene and eucalyptol, at a total concentration of 336 µg/mL but with different composition ratios. As shown in Table 4, the DPPH scavenging activity was significantly increased when curzerene and eucalyptol were mixed and showed higher DPPH scavenging activity than *C. wenyujin* Y.H. Chen et C. Ling essential oil. The result clearly revealed that a synergistic effect exists between curzerene and eucalyptol regarding to DPPH scavenging activity. Many studies reported the antioxidant synergistic effects between natural products’ extracts or compounds [40,41], however, to our best knowledge, this is the first time the antioxidant synergistic effect between curzerene and eucalyptol has been reported. In addition, in order to compare the DPPH scavenging activities of camphor, *δ*-elemene, and *β*-elemene, a higher concentration of these three compounds, 1.008 mg/mL, was tested for DPPH scavenging assay and their DPPH radical scavenging activities were 36.03%, 44.57%, and 39.07%, respectively.

In general, the DPPH scavenging activity ranking orders of these five compounds evaluated by the DPPH-GC offline method and DPPH radical scavenging assay were consistent by giving curzerene (peak 5) > eucalyptol (peak 1) > *δ*-elemene (peak 3) > *β*-elemene (peak 4) > camphor (peak 2), indicating that the DPPH-GC offline method can be used to qualitatively rank the antioxidant activity of the screened compounds from an essential oil. However, a high ratio of the decreased peak area of one screened compound does not necessarily represent that this compound has high DPPH scavenging activity, as indicated by the three weak active compounds *δ*-elemene (peak 3), *β*-elemene (peak 4), and camphor (peak 2). The potential reason leading to a significant decrease of peak areas of even weak antioxidants shown by the DPPH-GC offline method is that the high temperature in the GC machine facilitates the reaction of DPPH radicals and antioxidants. In addition, the DPPH-GC offline method is able to screen minor antioxidants from essential oil which are generally not detected by the TLC plate. Moreover, HSCCC is a desirable technique for combination with the DPPH-GC offline method for efficient separation of the potential antioxidants from essential oil by a target-guided manner.

## 4. Conclusions

In this study, a rapid and simple strategy for screening, identification, and separation of radical scavengers by off-line DPPH-GC-MS coupling HSCCC method was developed, and it was successfully applied for the investigation of potential antioxidants in *C. wenyujin* Y.H. Chen et C. Ling essential oil. Five compounds are preliminarily screened as antioxidants using the DPPH-GC method and then identified as eucalyptol (7.66%), camphor (2.34%), *δ*-elemene (1.15%), *β*-elemene (7.10%), and curzerene (15.77%) using GC-MS. Moreover, these five compounds are isolated by HSCCC using two solvent systems, *n*-hexane-acetonitrile-ethanol (5:3:2, *v/v*) and *n*-hexane-acetonitrile-acetone (4:3:1, *v/v*), and subjected to DPPH scavenging assay. Camphor, *δ*-elemene, and *β*-elemene show weak DPPH scavenging activity, while curzerene and eucalyptol show moderate DPPH scavenging activity. Notably, a significant synergistic effect on DPPH scavenging is found between curzerene and eucalyptol. The result demonstrated that off-line DPPH-GC-MS coupling CCC is an efficient method for screening, identification, and separation of antioxidant compounds in essential oil, which has advantages of high separation resolution, high detection sensitivity, easy structure identification, and target compound-oriented HSCCC separation. Thus, the newly developed method is expected to further promote the study on the evaluation of the antioxidant activity of the compounds in essential oils.

## Figures and Tables

**Table 1:** Scavenging activity of DPPH radical of the essential oil.

Sample	IC_50_ Values (μg/mL)
BHT	97.44 ± 2.63
Essential oil	292.88 ± 1.69

Each value in the table is the mean ± standard deviation (*n* = 3).

**Table 2:** Volatile compounds of *Curcuma wenyujin* Y.H. Chen et C. Ling essential oil identified by GC-MS.

NO.	Retention Time (min)	Compounds	Formula	Area Ratio (%)
1	8.18	α-Pinene	C_10_H_16_	0.51
2	8.66	Camphene	C_10_H_16_	0.94
3	9.58	*β*-Pinene	C_10_H_16_	0.49
4	11.4	Eucalyptol (peak 1)	C_10_H_18_O	7.66
5	15.93	Camphor (peak 2)	C_10_H_16_O	2.34
6	17.04	Isoborneol	C_10_H_18_O	0.84
7	17.47	Borneol	C_10_H_18_O	0.22
8	21.97	*δ*-Elemene (peak 3)	C_15_H_24_	1.15
9	23.89	*β*-Elemene (peak 4)	C_15_H_24_	7.1
10	25.08	Caryophyllene	C_15_H_24_	0.66
11	25.47	γ-Elemene	C_15_H_24_	0.51
12	26.24	Alloaromadendrene	C_15_H_24_	0.23
13	26.52	Humulene	C_15_H_24_	0.55
14	28.07	*β*-Eudesmene	C_15_H_24_	0.77
15	28.52	Curzerene (peak 5)	C_15_H_20_O	15.77
16	32.08	Guaiene	C_15_H_24_	0.24
17	35.16	Germacrone	C_15_H_22_O	2.4
18	36.02	7a-Isopropenyl-45-dimethyloctahydro-1H-indene-4-carboxylic acid	C_15_H_24_O_2_	6.23
19	38.88	*β*-Selinenol	C_15_H_26_O	2.85
20	39.41	Germacron	C_15_H_22_O	9.84
21	40.42	Curdione	C_15_H_24_O_2_	22.94
22	40.79	Aromadendrene oxide-(2)	C_15_H_24_O	1.67
23	41.56	Germacr-1(10)-ene-5,8-dione	C_15_H_24_O_2_	2.88
24	42.7	Ledene oxide-(II)	C_15_H_24_O	0.52
25	44.04	5,6-azulenedicarboxaldehyde, 1,2,3,3a,8,8a-hexahydro-2,2,8-trimethyl-, (3aα,8α,8aα)-(-)-	C_20_H_26_O_2_	1.24
26	44.26	Norethindrone	C_15_H_20_O_2_	0.53
27	45.26	Nootkaton-11,12-epoxide	C_15_H_22_O_2_	2.01
28	46.17	Deoxysericealactone	C_16_H_20_O_4_	1.09
29	47.43	Dehydrosaussurea lactone	C_17_H_24_O_4_	1.07
Total				95.23

**Table 3:** The partition coefficient values of compounds.

NO.	Solvent System	*K*-Value				
		Peak1	Peak2	Peak3	Peak4	Peak5
A	*n-*Hexane: acetonitrile: ethanol (5:3:2, *v/v*)	1.51	0.51	3.42	2.55	1.30
B	Petroleum ether: acetonitrile: acetone (4:3:1, *v/v*)	3.15	0.91	7.21	4.76	1.84

**Table 4:** Experimental scavenging capacity percentages (% ESC) for isolated compound from *Curcuma wenyujin* Y.H. Chen et C. Ling essential oil.

Sample	Concentration (µg/mL)	Eucalyptol/Curzerene Ratio, *w/w*	ESC (%)
Essential oil	3.36	-	68.98 ± 2.57 ^c^
Eucalyptol (1)	3.36	-	47.14 ± 6.62 *^d^*
Camphor (2)	10.08	-	36.03 ± 7.71 *^d^*
*δ*-Elemene (3)	10.08	-	44.57 ± 3.86 *^d^*
*β*-Elemene (4)	10.08	-	39.07 ± 5.45 *^d^*
Curzerene (5)	3.36	-	70.02 ± 5.86 *^b,c^*
mix 1	3.36	1:1	85.28 ± 3.83 *^a^*
mix 2	3.36	3:1	85.74 ± 3.70 *^a^*
mix 3	3.36	4:1	84.29 ± 2.58 *^a,b^*
mix 4	3.36	5:1	84.65 ± 4.24 *^a,b^*
mix 5	3.36	6:1	87.33 ± 2.77 *^a^*

Final concentration (µg/mL) of each antioxidant, each value was obtained by calculating the average of the three experiments ± standard deviation (*n* = 3). ESC (%) marked with different letters (*a*,*b*,*c*,*d*) were significantly different from each other (*p* < 0.05).
